# SARS-CoV-2 Is Not Detected in the Cerebrospinal Fluid of Encephalopathic COVID-19 Patients

**DOI:** 10.3389/fneur.2020.587384

**Published:** 2020-12-11

**Authors:** Dimitris G. Placantonakis, Maria Aguero-Rosenfeld, Abdallah Flaifel, John Colavito, Kenneth Inglima, David Zagzag, Matija Snuderl, Eddie Louie, Jennifer Ann Frontera, Ariane Lewis

**Affiliations:** NYU Grossman School of Medicine and NYU Langone Health, New York, NY, United States

**Keywords:** SARS-CoV-2, COVID-19, CSF, encephalopathy, cerebrospinal fluid

## Abstract

Neurologic manifestations of the novel coronavirus SARS-CoV-2 infection have received wide attention, but the mechanisms remain uncertain. Here, we describe computational data from public domain RNA-seq datasets and cerebrospinal fluid data from adult patients with severe COVID-19 pneumonia that suggest that SARS-CoV-2 infection of the central nervous system is unlikely. We found that the mRNAs encoding the ACE2 receptor and the TMPRSS2 transmembrane serine protease, both of which are required for viral entry into host cells, are minimally expressed in the major cell types of the brain. In addition, CSF samples from 13 adult encephalopathic COVID-19 patients diagnosed with the viral infection via nasopharyngeal swab RT-PCR did not show evidence for the virus. This particular finding is robust for two reasons. First, the RT-PCR diagnostic was validated for CSF studies using stringent criteria; and second, 61% of these patients had CSF testing within 1 week of a positive nasopharyngeal diagnostic test. We propose that neurologic sequelae of COVID-19 are not due to SARS-CoV-2 meningoencephalitis and that other etiologies are more likely mechanisms.

## Introduction

The novel coronavirus SARS-CoV-2, responsible for the COVID-19 pandemic, principally affects the respiratory system and, in severe cases, leads to pneumonia complicated by acute respiratory distress syndrome (ARDS). The viral infection impacts other organ systems as well and can cause gastrointestinal symptoms, renal insufficiency, and thrombosis among other complications. However, little is known about the neurologic manifestations and related mechanisms of COVID-19.

A common observation is that patients that develop severe COVID-19 pneumonia and ARDS manifest encephalopathy during the course of the disease and their recovery (1, 2). Possible etiologies for such central nervous system (CNS) impairment include direct infection of neural tissue, hypoxic ischemic injury, ischemic infarcts due to hypercoagulability, metabolic encephalopathy (including renal and hepatic etiologies), or effects of massive systemic cytokine release. A recent autopsy study of COVID-19 patients based on detection of viral genome by *in situ* hybridization suggested the brain abundance of the virus is approximately 10^6^-fold less than in the lung and 10^3^-fold less than in the kidneys, liver and heart (3). However, this low-level presence of viral genome in CNS tissue does not really address the question of whether the neurologic sequelae of COVID-19 are related to SARS-CoV-2 encephalitis/meningitis. Furthermore, it is not clear whether the patients in this autopsy study had any neurologic manifestations.

To help answer the question of whether SARS-CoV-2 causes meningoencephalitis in encephalopathic COVID-19 patients, we utilized a two-pronged approach. First, we asked whether brain tissue expresses the cell surface proteins required for viral entry into host cells, ACE2 and TMPRSS2 (4). And second, we tested whether SARS-CoV-2 RNA is detected in the cerebrospinal fluid (CSF) of encephalopathic COVID-19 patients.

## Methods

### Computational Analysis of RNA-seq Data

We analyzed public-domain RNA-seq data (both bulk and single-cell) from normal brain tissue in the GTEx, Brain RNA-seq and Allen Brain Atlas databases [www.brainrnaseq.org; celltypes.brain-map.org/rnaseq/human/cortex; (5)] for expression of *ACE2* and *TMPRSS2* mRNA. We used *GRIN2A*, which encodes the 2A subunit of the N-methyl-D-aspartate (NMDA) receptor and *GFAP* (glial fibrillary acidic protein) mRNAs as positive controls for expression in neurons and astrocytes, respectively.

### Validation of CSF Testing by SARS-CoV-2 RT-PCR

Current diagnostic modalities for COVID-19 rely predominantly on the detection of SARS-CoV-2 RNA using RT-PCR. Most clinical laboratories have implemented commercial platforms that have received Emergency Use Authorization (EUA) by the FDA. The advantage of commercial platforms is their reliability, well-standardized reagents, automation and high throughput. The extent of the pandemic and particularly its impact in New York City made these platforms a highly desirable option. Our institution used the Cepheid (Sunnyvale, CA) Xpert® Xpress, as well as the Roche (Basel, Switzerland) Cobas®, platforms for SARS-Cov-2 RT-PCR by nasopharyngeal swab. Both systems have comparable performance with similar limits of detection (LOD), 250 and 100–200 RNA copies/mL, respectively, which is important for consistency of results.

As clinical demands for CSF testing for SARS-CoV-2 RT-PCR testing in our institution increased, we planned the validation of this sample type according to the New York State Wadsworth Center procedure for SARS-CoV-2 Laboratory Developed Test (LDT). The Food and Drug Administration (FDA) has given the NYS Wadsworth Center the authority to approve SARS-CoV-2 LDT tests to qualified laboratories during the COVID-19 pandemic.

We chose the Cepheid Xpert® Xpress platform, which targets the *N2* (nucleocapsid) and *E* (envelope) sequences of the SARS-CoV-2 genome, to validate SARS-CoV-2 RT-PCR in CSF specimens. Detection of the SARS-CoV-2-specific *N2* sequence results in a positive diagnostic test. Refrigerated CSF samples, obtained from patients without COVID-19 and previously analyzed for other tests in the laboratory, were pooled and used as the sample matrix for validation studies. The pooled CSF tested negative by SARS-CoV-2 RT-PCR. To determine the limit of detection (LOD), we added to the pooled CSF serial 2-fold dilutions of SeraCare AccuPlex™ Reference Material (Cat # 0505-0126), which contains 5,803 copies/mL of SARS-CoV-2 RNA, including the target *N2* sequence detected by the Cepheid Xpert Xpress platform. The lowest dilution of reference material that we tested was 90 copies/mL. All dilutions were tested in triplicates. We determined the LOD in CSF to be 181 copies/mL, comparable to the platform's LOD in nasopharyngeal swab specimens. This LOD was 100% reproducible in 20 replicate experiments.

To determine the specificity of the assay, we spiked our pooled CSF with control material containing viral genomic sequences of cytomegalovirus (CMV), herpes simplex virus 1 (HSV1), herpes simplex virus 2 (HSV2), parechovirus type 3, enteric cytopathic human orphan (ECHO) virus, and human herpesvirus 6 (HHV6). We also included CSF proficiency panel samples containing HSV1 and CMV, as well as one clinical CSF sample known to contain HSV1. All these samples tested negative for SARS-CoV-2 by RT-PCR.

To further evaluate the assay for clinical use, we used contrived individual CSF samples spiked with Seracare AccuPlex™ Reference Material at six different increments of the LOD (2X, 4X, 6X, 8X, 10X, and 25X). We included five CSF samples per condition. Ten unspiked CSF samples were used as negative controls. All 30 contrived samples spiked with different LOD increments tested positive by SARS-CoV-2 RT-PCR, while all 10 controls tested negative.

### Retrospective Analysis

To test if SARS-CoV-2 can be detected in the CSF of COVID-19 patients with neurologic complications, we retrospectively mined data from our COVID-19 adult patient cohort in the NYU Langone Health System in New York City (NYC) and searched for SARS-CoV-2 RT-PCR tests in CSF.

## Results

**Figure 1:**
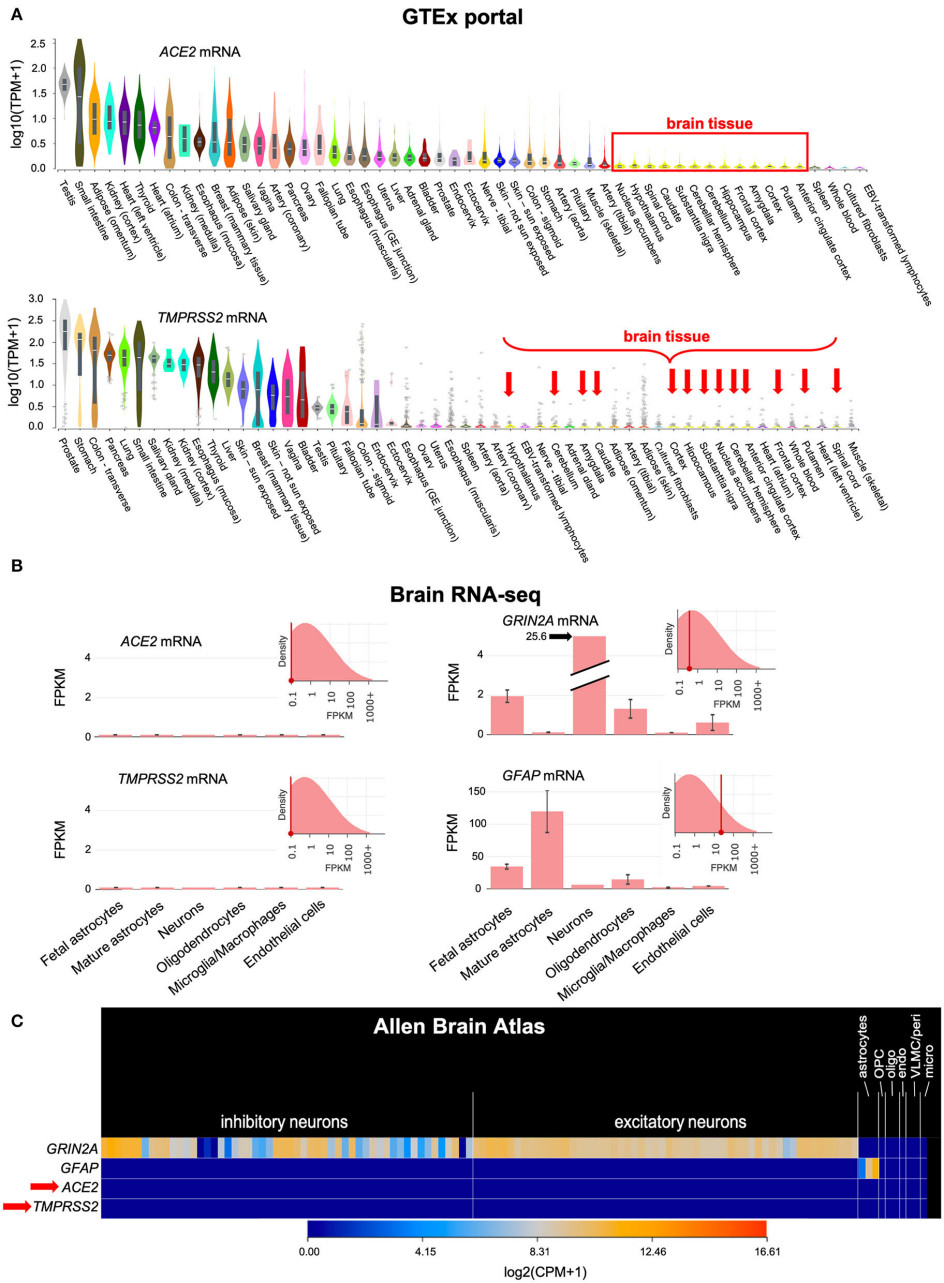
*ACE2* and *TMPRSS2* mRNAs are only minimally expressed in the brain. **(A)** Bulk RNA sequencing of normal human tissues in the GTEx portal shows that *ACE2* and *TMPRSS2* mRNAs are minimally expressed in the human brain (red box and arrows). **(B)** The Brain RNA-seq database, which is based on RNA-seq of sorted cellular populations, shows minimal expression of *ACE2* and *TMPRSS2* in brain cells, including neurons, glia, microglia and endothelial cells. The expression pattern of *GRIN2A*, which encodes the NMDA receptor 2A subunit in neurons, and *GFAP*, which encodes an intracellular filament found in astrocytes, are shown for comparison. **(C)** Similar information is found in the Allen Brain Atlas, which is based on single-cell RNA-seq of normal brain specimens. *ACE2* and *TMPRSS2* (red arrow) are minimally expressed. *GRIN2A* and *GFAP* are again shown for comparison. TPM, Transcripts Per Million mapped reads; FPKM, Fragments Per Kilobase of transcript per Million mapped reads; CPM, Counts Per Million mapped reads; OPC, Oligodendrocyte Precursor Cells; oligo, oligodendrocytes; endo, endothelial cells; VLMC/peri, Vascular and LeptoMeningeal Cells/pericytes; micro, microglia.

SARS-CoV-2 requires the cellular receptor ACE2 and the transmembrane serine protease TMPRSS2 to enter cells (4). Our analysis of public-domain RNA-seq data indicates that only minimal amounts of *ACE2* and *TMPRSS2* mRNAs are expressed in human brain cells, including neurons, glia, microglia and endothelial cells (Figure 1). This suggested that the brain may be less susceptible to infection than other tissues with higher expression of ACE2 and TMPRSS2.

**Figure 2:**
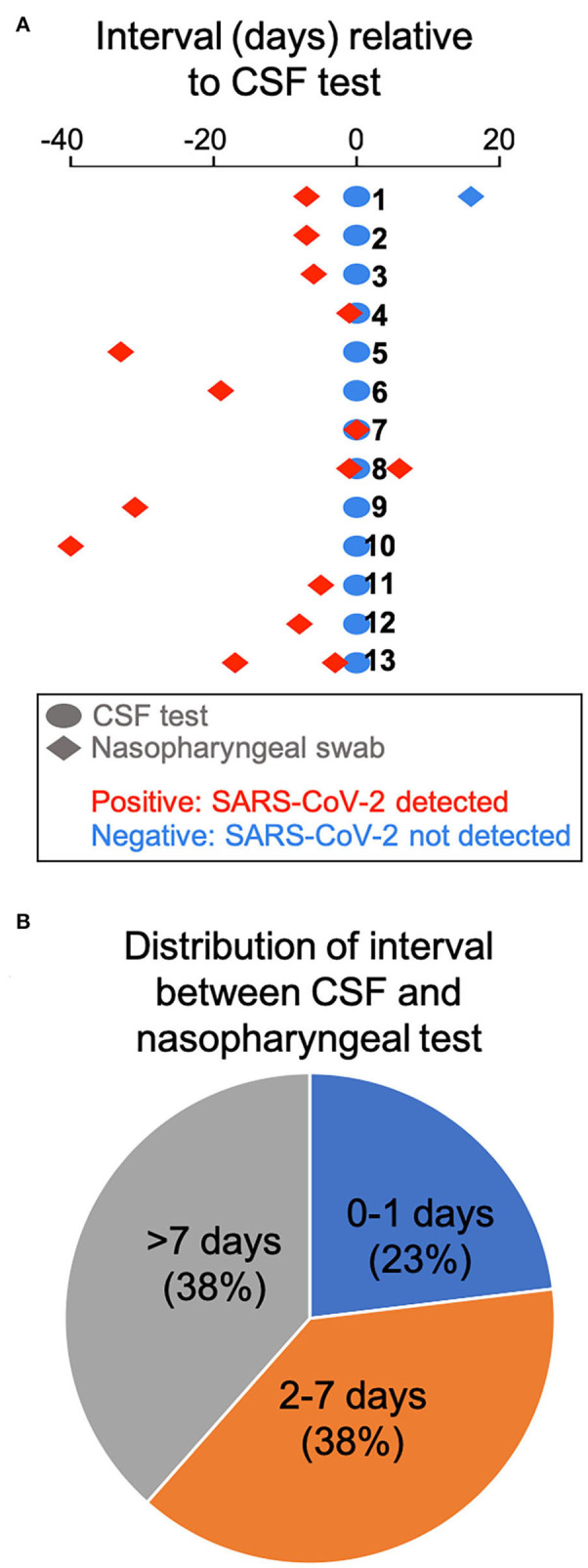
Time interval between COVID-19 diagnosis by nasopharyngeal swab and CSF testing. **(A)** The interval between CSF testing and the nasopharyngeal swab test is displayed for all 13 patients in the cohort. **(B)** Pie chart showing the distribution of interval between CSF and nasopharyngeal testing.

We retrospectively identified 13 adult patients with severe COVID-19 pneumonia (Table 1) who had CSF tested for SARS-CoV-2 by RT-PCR, due to concern for meningoencephalitis. CSF sampling was obtained via lumbar puncture (*n* = 11), by tapping the reservoir of a ventriculoperitoneal shunt valve (n=1), and by aspirating CSF through an external ventricular drain (EVD) (*n* = 1). Indications included encephalopathy (*n* = 7), seizures (*n* = 4), and encephalopathy associated with known intracranial hemorrhage (*n* = 2). Encephalopathic symptoms preceded CSF sampling in all cases. All patients had SARS-CoV-2 infection confirmed by nasopharyngeal swab testing, but, importantly, none showed evidence of the virus in the CSF by RT-PCR. There was no pleocytosis in the CSF, with the exception of 1 patient with known subarachnoid hemorrhage (Table 1). Figure 2A shows the time interval between the nasopharyngeal and CSF tests in these patients. Of particular interest are three patients (23.1%) whose CSF was tested the same day (*n* = 1) and the day after (*n* = 2) the nasopharyngeal swab that indicated SARS-CoV-2 infection (Figure 2B). In one of these cases, a second nasopharyngeal swab 8 days after the CSF test remained positive for the virus. Overall, 53.8% of patients in this cohort had their CSF tested within 1 week after initial diagnosis; and 61.5% had the CSF test within 1 week of a positive nasopharyngeal swab test (Figure 2B). Eleven of the patients (84.6%) had brain imaging (MRI or CT) at the time of the CNS workup, with nine of those (81.8%) showing abnormal findings. These imaging abnormalities included, in at least three cases, evidence for subcortical hypoxic ischemic injury and infarcts.

**Table 1:** Patient demographics and clinical information.

**Patient number**	**Sex**	**Age (years)**	**CSF test date**	**Outcome**	**Method**	**Swab test date**	**Outcome**	**Interval (days)**	**Indication for CSF test**	**CSF glucose**	**CSF protein**	**CSF RBC**	**CSF WBC**	**CSF differential**	**Brain imaging**	**Admission date**	**Discharge date**	**Clinical outcome**	**Repeat swab test date**	**Outcome**
1	F	68	12/04/2020	Neg	LP	05/04/2020	Pos	7	Encephalopathy	65	37	0	3	80% lymphs	CT 4/5/20: normal	05/04/2020	30/04/2020	Skilled nursing facility	28/04/2020	Neg
2	F	66	27/03/2020	Neg	LP	20/03/2020	Pos	7	Encephalopathy	93	80	1	9	57% lymphs	No	20/03/2020	09/04/2020	Death		
3	M	88	20/04/2020	Neg	LP	14/04/2020	Pos	6	Seizure	64	62	0	14	99% lymphs	CT 4/14/20: abnormal hypodensities	14/04/2020	28/04/2020	Recovered		
4	M	69	25/03/2020	Neg	LP	24/03/2020	Pos	1	Seizure	81	86	14,000	24	75% PMNs	CT, CTA 3/24/20, 3/26/20: lacunar infarct	24/03/2020	01/04/2020	Death		
5	M	38	26/04/2020	Neg	LP	24/03/2020	Pos	33	Encephalopathy	125	16	1	0		MRI 4/21/20, 4/29/20: hypoxic injury/infarcts	23/03/2020	10/06/2020	Rehabilitation		
6	F	22	20/04/2020	Neg	Shunt tap	01/04/2020	Pos	19	Encephalopathy	146	128	0	2		No	01/04/2020	12/05/2020	Death		
7	M	38	29/04/2020	Neg	LP	29/04/2020	Pos	0	Seizure	75	60	0	5	60% lymphs	CT 4/29/20: normal; MRI 5/1/20: normal	29/04/2020	08/05/2020	Recovered		
8	F	60	31/03/2020	Neg	LP	30/03/2020	Pos	1	Encephalopathy	34	85	13,000	31	78% lymphs	MRI brain 3/17/20, 3/27/20, 4/6/20: FLAIR hyperintensities	15/03/2020	10/04/2020	Recovered	06/04/2020	Pos
9	F	63	30/04/2020	Neg	LP	30/03/2020	Pos	31	Encephalopathy	62	25	1	0		MRI 4/29/20: FLAIR hyperintensities	30/03/2020	03/06/2020	Recovered		
10	M	64	05/05/2020	Neg	LP	26/03/2020	Pos	40	Encephalopathy	70	49	900	2		CT 4/23/20: normal; MRI 5/1/20: diffusion restriction and FLAIR hyperinsities	26/03/2020	22/05/2020	Recovered		
11	F	34	14/05/2020	Neg	LP	09/05/2020	Pos	5	Seizure	114	21	40	1	100% lymphs	CT 4/12: pons hypodensities	09/05/2020	30/06/2020	Recovered		
12	F	36	15/05/2020	Neg	EVD	07/05/2020	Pos	8	Hemorrhage/encephalopathy	62	219	46,000	50	69% PMNs	CT 5/13: hemicraniectomy for ICH, IVH	07/05/2020	08/07/2020	Hospice care		
13	F	67	12/05/2020	Neg	LP	25/04/2020	Pos	17 (3)	Hemorrhage/encephalopathy	53	153	2,000	402	78% PMNs	CT 5/12: ICH, SAH	25/04/2020	24/05/2020	Death	09/05/2020	Pos

*F, female; M, male; neg, negative; pos, positive; LP, lumbar puncture; EVD, external ventricular drain; RBC, red blood cells; WBC, white blood cells; lymphs, lymphocytes; PMNs, polymorphonuclear cells; ICH, intracranial hemorrhage; IVH, intraventricular hemorrhage; SAH, subarachnoid hemorrhage*.

## Discussion

Although this is a small cohort of patients and the data were analyzed retrospectively, our negative CSF findings are consistent with the observation that *ACE2* and *TMPRSS2* mRNAs are minimally expressed in the brain. We postulate that SARS-CoV-2 is not likely to establish persistent or severe meningoencephalitis that can account for the neurologic sequelae of COVID-19, a hypothesis that will have to be tested in larger studies in the future. In theory, possible routes of spread of the novel coronavirus to the brain could include: viremic spread and infection of cerebrovascular endothelium or diapedesis through the vasculature or blood-brain barrier; the choroid plexus, the tissue responsible for CSF production; transsynaptic spread to the brain via the olfactory epithelium in the nasal cavity; or introduction of the virus by infected peripheral leukocytes, in a “trojan horse” scenario. However, we believe that, even if SARS-CoV-2 were to reach the brain via one of these mechanisms, it would nonetheless be unable to establish a clinically significant, persistent infection due to minimal expression of ACE2 cellular receptor and TMPRSS2 transmembrane protease, both necessary for viral entry into cells (4). Ultimately, autopsy studies of such encephalopathic COVID-19 patients will be required to determine whether SARS-CoV-2 infects brain tissue, using techniques such as electron microscopy, which identifies viral particles; *in situ* hybridization for the viral genome; or immunohistochemistry for viral proteins (3).

These observations need to be considered in the context of an isolated report of SARS-CoV-2 meningitis/cerebritis (albeit with a negative nasopharyngeal test result) (6), which suggests that CNS infection may be possible. However, recent studies corroborate our findings that SARS-CoV-2 is not detected in the CSF of COVID-19 patients (2, 7). Our case series, one of the largest so far on the status of CSF in COVID-19 patients, builds on the prior reports to more definitively argue against SARS-CoV-2 meningoencephalitis, because it utilizes a CSF-validated RT-PCR diagnostic assay, and, equally importantly, because it reports negative CSF testing within 0–1 days from COVID-19 diagnosis in 23.1% of patients, and within 1 week of positive nasopharyngeal testing in 61.5% of the subjects. The results of early CSF testing in these patients essentially fulfill the criterion for near-simultaneous nasopharyngeal swab and CSF assays during the critical window of active infection, in order to safeguard against the concern that a long delay in CSF testing after initial diagnosis may be negative anyway due to viral clearance.

Limitations of our study include the small sample size, as well as the possibility that low-grade CSF infection with SARS-CoV-2 may fall below the limit of detection of our diagnostic test. Additional patients tested after this manuscript was written also tested negative for SARS-CoV-2 in the CSF. Larger studies will be required to ascertain our findings in this small cohort.

Overall, our study suggests that SARS-CoV-2 infection of the CNS is unlikely to account for neurologic symptoms of the COVID-19 syndrome. In our opinion, the CNS manifestations of COVID-19 are more likely the result of a coagulation disorder leading to multiple ischemic infarcts; hypoxic ischemic injury; toxic metabolic effects of prolonged critical illness, residual sedation and uremic encephalopathy; or CNS effects of the cytokine storm that characterizes severe COVID-19 cases (1, 2, 8).

## Data Availability Statement

Publicly available datasets were analyzed in this study. This data can be found here: www.brainrnaseq.org and celltypes.brain-map.org/rnaseq/human/cortex.

## Ethics Statement

The studies involving human participants were reviewed and approved by NYU School of Medicine IRB. Written informed consent for participation was not required for this study in accordance with the national legislation and the institutional requirements.

## Author Contributions

DP, EL, JF, and AL provided clinical data. MA-R, AF, JC, KI, DZ, and MS validated the SARS-CoV-2 PCR assay in CSF. DP wrote the manuscript, which was edited by all other authors. All authors contributed to the article and approved the submitted version.

## Conflict of Interest

The authors declare that the research was conducted in the absence of any commercial or financial relationships that could be construed as a potential conflict of interest.
